# Access for sale? Overlying rights, land transactions, and groundwater in California

**DOI:** 10.1088/1748-9326/ad0f71

**Published:** 2024-01-23

**Authors:** Jenny Linder Rempel, Ella Belfer, Isha Ray, Rachel Morello-Frosch

**Affiliations:** 1 Energy & Resources Group, University of California, Berkeley, CA, United States of America; 2 Department of Environmental Science, Policy, and Management, University of California, Berkeley, CA, United States of America; 3 School of Public Health, University of California, Berkeley, CA, United States of America

**Keywords:** groundwater, land use, water access, agriculture, wells

## Abstract

Climate change intensifies longstanding tensions over groundwater sustainability and equity of access among users. Though private land ownership is a primary mechanism for accessing groundwater in many regions, few studies have systematically examined the extent to which farmland markets transform groundwater access patterns over time. This study begins to fill this gap by examining farmland transactions overlying groundwater from 2003–17 in California. We construct a novel dataset that downscales well construction behavior to the parcel level, and we use it to characterize changes in groundwater access patterns by buyer type on newly transacted parcels in the San Joaquin Valley groundwater basin during the 2011–17 drought. Our results demonstrate large-scale transitions in farmland ownership, with 21.1% of overlying agricultural acreage statewide sold at least once during the study period and with the highest rates of turnover occurring in critically overdrafted basins. By 2017, annual individual farmland acquisitions had halved, while acquisitions by limited liability companies increased to one-third of all overlying acres purchased. Together, these trends signal increasing corporate farmland acquisitions; new corporate farmland owners are associated with the construction, on comparable parcels, of agricultural wells 77–81 feet deeper than those drilled by new individual landowners. We discuss the implications of our findings for near-term governance of groundwater, and their relevance for understanding structural inequities in exposure to future groundwater level declines.

## 1. Introduction

Groundwater is a vital resource, providing an estimated 43% of global irrigation water [1] and drinking water for half of the world’s population [2]. Climate change is likely to affect both the timing and magnitude of groundwater withdrawal and recharge, raising concerns about groundwater quality and supply [3, 4] and inequities in access [5]. In California, historic and persistent drought conditions over the last two decades have sharply increased pressure on groundwater resources [6, 7]. The prospect of more frequent and severe droughts [3, 8] makes it essential to understand who can access groundwater and how.

Where institutions bundle land and groundwater rights, questions of access extend to the land surface overlying groundwater basins. By access, we refer to the ability to benefit from groundwater [9]. In states that manage groundwater through absolute ownership or correlative rights regimes, land and groundwater rights are legally intertwined: ownership of land directly confers access to groundwater [10]. California’s historic 2014 Sustainable Groundwater Management Act (SGMA) enables overlying groundwater rights-holders to trade water rights and participate in subbasin scale groundwater governance, but it does not legally alter the water rights regime (Cal. Water Code § 10720.5): most private users must acquire overlying land to obtain groundwater rights [11]. Accordingly, the private land market has a substantial but underexplored role in determining groundwater access. Despite concerns about the impacts of large-scale land acquisitions on equitable and sustainable groundwater access [12–14], we are not aware of any systematic examination of land market effects on groundwater access.

In general, references to overlying land acquisitions are rare in groundwater scholarship ([15] is a notable exception), and farmland market studies usually do not distinguish between land transactions on overlying and non-overlying land, e.g. [16–19]. While political ecology studies underscore the bi-directional relationship between land and groundwater, e.g. [20–22], the combination of limited downscaled groundwater data and limited land transactions data makes empirical analyses of these relationships a challenge [15]. In this paper we ask: if groundwater influences land markets, how might land markets affect groundwater access?

Attention to farmland turnover can reveal corresponding changes in groundwater access patterns and user characteristics. Though farmland is considered an illiquid asset, increases in farmland transfers are anticipated across the US as farmers retire [16, 23]. Farmland sales are a key mechanism through which, over relatively short timescales, the characteristics of new buyers may change at scale, with implications for both land and water management strategies [24–26]. The connection between landowner characteristics and surface water management practices is well-established [27–29], and a limited number of studies suggest similar connections with groundwater access and management strategies [15, 30–32]. Despite longstanding concerns about the unevenness of regional well deepening trends [33–35], groundwater research has little to say on those who acquire, manage, and use it [36, 37]. Within California, the extent and rapidity of overlying farmland turnover—and corresponding effects on groundwater rights and access—remain understudied.

With national decreases in individual farmland ownership and the rising prevalence of entities such as limited liability companies (LLCs), farmland turnover may not only alter farmer preferences, but more fundamentally reconfigure the legal landscape of farmland ownership [16, 38, 39]. As [40] point out, the question is not only who, but *‘what’* acquires land. Several scholars have raised concerns about the environmental and social impacts of new financial and corporate vehicles for farmland acquisitions [38, 39, 41]. Yet their current and future impacts on groundwater access and use are understudied. In California, recent case studies indicate that the emergence of new types of buyers may have negative groundwater consequences, such as neighboring domestic well failures [42], the pursuit of less stringent regulatory targets for groundwater depth levels [14], and the growing influence of privately-controlled water banks on groundwater access [43]. To the extent that these cases reflect larger-scale transitions in buyer types and behaviors, they signal shifts with potentially transformative implications for land and water management.

This analysis elucidates the role of California’s farmland market in determining groundwater access. We construct a novel dataset that downscales well construction behavior to the parcel level, linking land transactions and well construction records from 2003–2017 to: (1) characterize arm’s-length farmland turnover trends (i.e. independent market transactions, rather than inheritance or intra-family transfers) that could have adverse implications for groundwater sustainability and equity of access; and (2) test the implications of landowner type on the depth of agricultural wells built on recently purchased parcels in the San Joaquin Valley during the 2011–17 drought. As California’s groundwater faces increasing socio-environmental stressors, this study can inform near-term groundwater decision-making, with relevance to other drought-prone regions where land and water rights are bundled.

## 2. Methods

**Figure 1.**
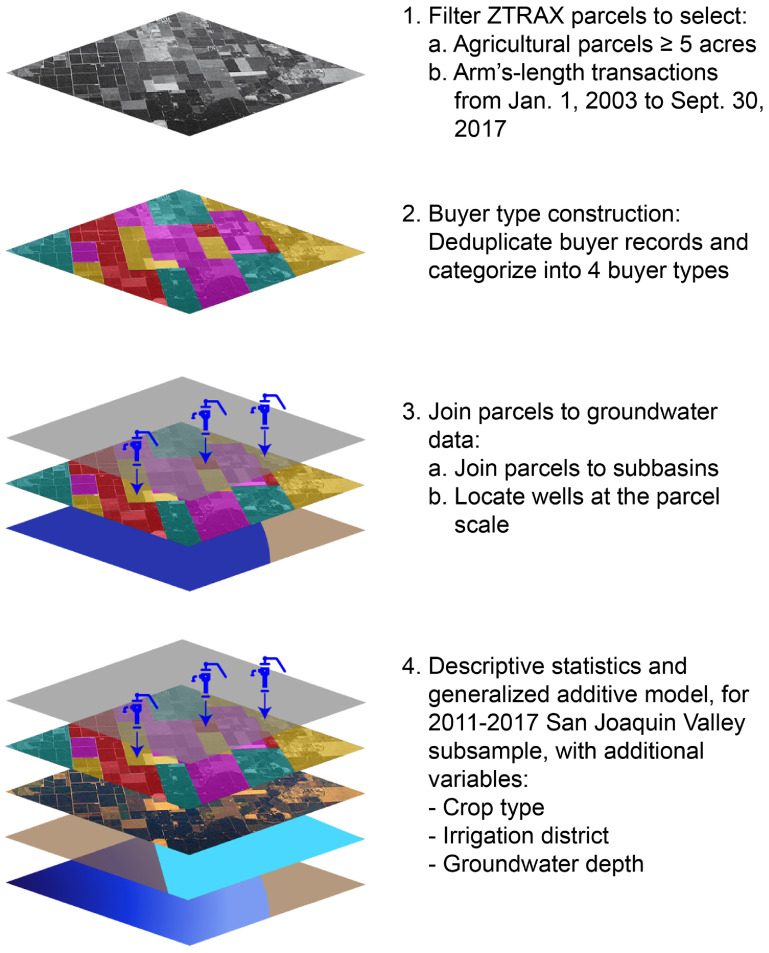
The workflow for our four-step method to integrate information on well depth, transacted farmland parcels, and buyer types. Numeric entries indicate the four major processing steps, and alphabetic entries indicate key sub-steps. *Note.* Image from *California’s Central Valley* by [44]. CC BY SA 3.0. ‘Hand pump’ symbol by Vectors Market from thenounproject.com.

We developed a four-step workflow to integrate information on well depth, transacted farmland parcels, and buyer types (figure 1 and supplementary material, SM).

### 2.1. Identifying relevant transacted parcels

We characterized farmland and groundwater access trends using the ZTRAX^®^ dataset, which contains transaction and ownership information based on existing cadastral data [45]. Our analysis included all arm’s-length transactions (i.e. non-distressed sales with a deed type and price that did not reflect non-market transfers such as inheritance or intra-family transfers) of parcels $ \unicode{x2A7E} 5$ acres and designated as agricultural or rural residential land between 1 January 2003 and 30 September 2017 (S1: Identifying Relevant Transacted Parcels). To link ZTRAX assessment and transactions data, we used unique identifiers generated by Zillow [45], as well as a Jaro-Winkler algorithm to match addresses with a string distance threshold of 0.9 [46]. Finally, we joined ZTRAX data to a 2018 SmartParcels^®^ parcel shapefile dataset [47] and removed all parcels with non-agricultural land use codes. The final merged dataset contains 89 597 transactions of 79 699 unique parcels of interest.

### 2.2. Buyer type construction

We used string distance algorithms to link parcels and transactions associated with the same buyers [46, 48]. We separated buyer records into individual (*n* = 155 421) and non-individual (*n* = 62 129) categories, as determined by ZTRAX, and matched records within each category by buyer name and address, using a minimum string distance threshold of 0.9.

Based on our literature review, we constructed four mutually exclusive farmland buyer categories: LLCs, non-LLC companies, individuals, and family investors (S2: Buyer Type Construction). We focus on LLCs as a relatively new corporate farmland acquisition type [19, 49], and we include family investors given the long-standing practice of shared farmland ownership among family members and, increasingly, their affiliated companies [16, 41, 50, 51]. To identify family linkages with trusts, IRAs, and businesses, we built on the literal-legal method [52], which links land ownership records based on the legal names on the title. Ultimately, we identified 75 353 buyer groups, of which 54 734 are individuals, 6598 are family investors, 5374 are LLCs and 8647 are non-LLC companies.

### 2.3. Groundwater and well construction data

We defined any parcel that at least partially overlies one of the 515 alluvial subbasins monitored by the California Department of Water Resources (CADWR) as an overlying parcel [53], and we classified basins as adjudicated, high priority, and critically overdrafted following state designations [54] (S3: Groundwater and Well Construction Data). Given the absence of groundwater withdrawal data in most groundwater basins and the salience of well depth to groundwater access [55–57], the depth of newly constructed agricultural wells is used as a proxy for changes in groundwater access on transacted parcels [15].

To identify newly constructed wells, we used California’s Online System for Well Completion Reports, which includes data on well locations, constructed depths, and use types [58]. Drawing on [59], we retained agricultural wells built between 1 January 2003 and 30 September 2017 to match the timeframe of parcel transaction data. We used five methods to match wells to both transacted and non-transacted parcels located within a half-mile buffer of Public Land Survey System sections, leveraging the assessor’s parcel number, a Jaro-Winkler distance algorithm on well and parcel addresses, and the Google Geocoding API. Of the 15 348 wells retained for matching, we matched 3315 (21.6%) to transacted and 7376 (48.1%) to non-transacted parcels. We removed from analysis wells matched to parcels that were not overlying alluvial subbasins and wells built prior to parcel purchase. A total of 2462 wells built on recently transacted parcels were retained.

To examine relationships between landowner type and well depth, we used a subsample of the 2003–2017 well dataset. To address large-scale variation in surface water deliveries between drought and non-drought years, we selected wells constructed during the 2011–2017 drought on parcels sold since 1 January 2003. Based in part on the regional availability of groundwater depth contours, we further reduced the dataset to include wells built in the San Joaquin Valley (SJV) groundwater basin, retaining 1603 wells for analysis. Groundwater sustainability and equity concerns are particularly pronounced in the arid SJV basin (figure S4.3) [60–63], which is comprised almost entirely of critically overdrafted groundwater subbasins. The SJV basin is a clastic sedimentary alluvial aquifer-aquitard complex that primarily operates in a semi-confined manner with downward-oriented vertical hydraulic gradients [64, 65].

We interpolated depth to groundwater from the annual spring groundwater depth contours produced by CADWR, using the Triangulated Irregular Network method [53, 66, 67], and we assigned each well the mean groundwater elevation across the entire parcel during the preceding spring (e.g. spring 2015 for a well built in October 2015). Well records with incomplete data on drill depth, construction date, or groundwater depths were removed, with 1179 wells retained for analysis (table S3.3).

### 2.4. Data analysis

We calculated descriptive statistics for well depth, buyer type, and parcel characteristics. We then analyzed the association between well depth and the buyer type of landowners who drill wells on recently purchased agricultural parcels, controlling for covariates, including parcel size, year of well construction, depth to groundwater, surface water availability, crop type, and well coordinates. We constructed surface water availability as a binary indicator: any parcel located within the boundaries of a water district receiving agricultural irrigation water was marked as having potential access to surface water [68–70]. Crop types were categorized as orchard, vineyard, or row crops/pasture using USDA Cropland Data, following [71]. Using Macaulay and Butsic’s [72] scheme, parcels were assigned to the crop type covering the most parcel area in the well construction year.

We estimated associations between well depth, buyer type, and parcel characteristics using generalized additive models (GAMs). The equation is as follows:

\begin{align*}{{Y = }}{{{\beta }}_{\text{0}}}{{ + }}{{{\beta }}_{\text{1}}}\left( {{{{x}}_{\text{1}}}} \right){{ + }}{{{\beta }}_{\text{2}}}\left( {{{{x}}_{\text{2}}}} \right){{ + \ldots + }}{{{f}}_{{m}}}\left( {{{{x}}_{{m}}}} \right) +\varepsilon\end{align*}

where *Y* is the dependent variable (i.e. well depth), *x_i_* are predictor variables (e.g. groundwater depth, crop type), and *f_m_* is a smooth function [73]. In this instance, to account for spatial autocorrelation between neighboring wells, we fit a spline smoothing function on the well coordinates. This smoothing technique has been applied to adjust for spatial autocorrelation in similar studies [74]. Moran’s Index (Moran’s I) was used to test the spatial autocorrelation of residuals [75]. Model fit was assessed using autocorrelation function (ACF) and partial ACF (PACF) plots; quantile–quantile (Q–Q) plots; a plot of the residuals versus the fitted values; the Akaike information criterion (AIC); and Log Likelihood.

We ran two multivariate models. First, we tested whether each buyer type was associated with different well depths, with individual buyers as the comparison group. Second, we focused on company buyer types to assess whether LLCs and family investors, which are both relatively recent farmland owner types, are associated with different well depths compared to non-LLC companies.

## 3. Results

We present our results in three parts. First, we assess the scale of farmland transactions, characterizing the extent of turnover on overlying land and within different types of groundwater basins. Second, we characterize temporal changes in buyer type and by groundwater basin type. Finally, we present our results on the association between buyer types and the depths of newly constructed wells.

### 3.1. Farmland turnover rates

Between 1 January 2003 and 30 September 2017, 18.1% of all agricultural acreage in California was transacted at least once, with a 2.0% average annual turnover rate statewide (table 1; also figure S4.1 for sales over time). Critically overdrafted basins experienced the highest rates of land turnover, with 23.5% of agricultural acreage sold at least once over the study period.

**Table 1.** Farmland turnover characteristics by region, January 2003–September 2017.

Region	Agricultural acres bought	Percentage of agricultural acreage bought at least once	Average annual turnover rate (acreage)^a^
Statewide	5058 127	18.1%	2.0%
Land Overlying Alluvial Basins	3149 321	21.1%	2.4%
High Priority Basins^b^	1979 429	22.4%	2.5%
Critically Overdrafted Basins^c^	1503 446	23.5%	2.6%
Adjudicated Basins^d^	148 808	19.7%	2.4%
Land Not Overlying Alluvial Basins	1908 806	14.7%	1.7%

^a^ Calculated as the total number of unique acres belonging to a region transacted in a calendar year, divided by the total number of acres in that region. Any parcel transacted more than once in a calendar year was only counted once. These annual turnover rates are then averaged over the timeframe of analysis.

^b^ High prioritization indicates a high degree of importance of groundwater to overlying populations, agriculture and ecosystems (Statewide Groundwater Elevation Monitoring Program, Cal. Water Code § 109339(b)).

^c^ Critically overdrafted basins are basins in which current water management practices, if continued, would likely have adverse environmental, social, or economic impacts [76].

^d^ In adjudicated basins, all groundwater users’ rights have been defined and determined as a result of past legal rulings [77].

**Figure 2.**
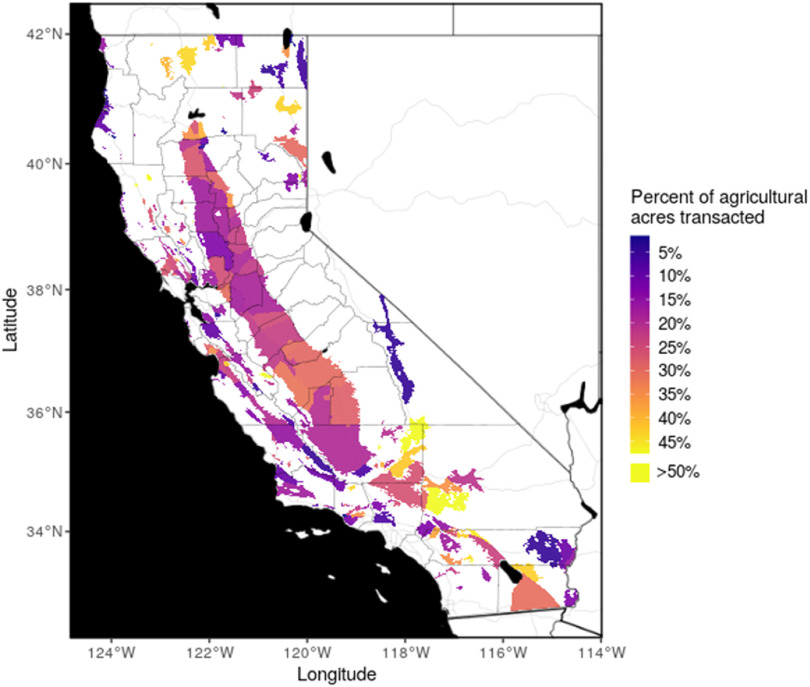
Percent of unique overlying acreage transacted at least once between 2003–17 by subbasin with >5 farmland parcels transacted, with county boundaries in black for reference.

The extent of farmland turnover varied widely by subbasin across the state (figure 2). In the SJV basin, 23.5% of agricultural acreage changed hands.

### 3.2. Changes in farmland buyer type

**Figure 3.**
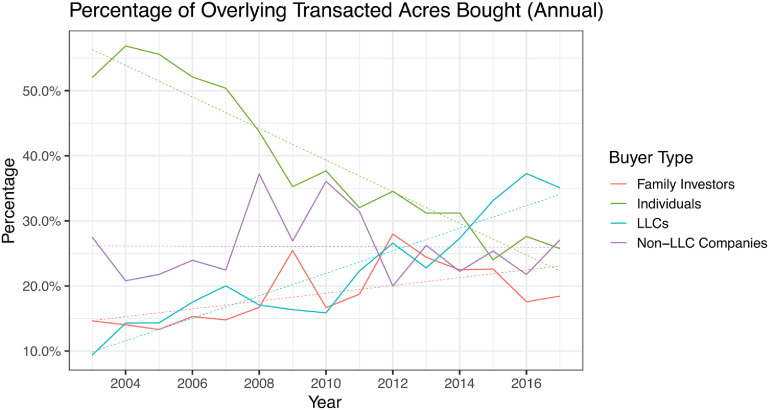
Percentage of transacted acres overlying alluvial basins bought annually by buyer type, from January 2003–September 2017. Linear trendlines added for visual interpretation.

The composition of buyers acquiring overlying farmland changed substantially from 2003–17 (figure 3). The percentage of overlying acres bought annually by individual farmland buyers declined sharply, from 52.0% to 25.7%, and was not offset by the number of acres acquired by family investors. By the end of the study period, more than one third of all agricultural acres bought in any given year were purchased by LLCs.

Table 2 shows that from 2003–17, LLCs on average bought 5.7 times as many acres of overlying farmland statewide (192 acres) as individual buyers (34 acres). Within critically overdrafted basins statewide, the average LLC bought 6.9 times as many acres as the average individual buyer.

**Table 2.** Mean agricultural acreage acquisitions for each farmland buyer statewide, by buyer type, January 2003–September 2017, based on the final parcel owner (Standard Deviation—SD).

Buyer	Individuals (*n* = 39 500)	Family investors (*n* = 5357)	LLCs (*n* = 4118)	Non-LLC companies (*n* = 5527)
All Acreage Bought	59 (SD = 235)	182 (SD = 616)	304 (SD = 1223)	215 (SD = 896)
Overlying Acreage Bought	34 (SD = 137)	121 (SD = 384)	192 (SD = 715)	155 (SD = 711)
High Priority Acreage Bought	20 (SD = 108)	83 (SD = 336)	116 (SD = 463)	109 (SD = 655)
Critically Overdrafted Acreage Bought	14 (SD = 91)	63 (SD = 281)	97 (SD = 449)	82 (SD = 620)

### 3.3. Well construction patterns on transacted parcels

Overlying farmland purchases resulted in active use of groundwater rights through agricultural well construction. Of the estimated 16 519 overlying agricultural wells built between 2003–17, we conservatively estimate 14.9% (*n* = 2462) were built following a farmland parcel transaction during that time. Between 2011–17 in the SJV, 25.0% of new agricultural wells were built on parcels after a 2003-17 sale (*n =*1603 of 6419). Distinctive local trends are observed (table S4.1).

From 2011–17 in the SJV, individual owners built most wells on transacted parcels (*n* = 511), and on average drilled shallower wells (mean 465 feet, SD 242) than all other buyer types (table 3). On average, LLCs drilled the deepest wells (mean 736 feet, SD 447) and drilled wells in locations with the greatest depth to groundwater (mean 154 feet, SD 114). Across buyer types, the proportion of acreage devoted to each crop type did not vary substantially (figure S4.2).

**Table 3.** Parcel and well characteristics for wells drilled on transacted parcels in the San Joaquin Valley groundwater basin, by buyer type (2011–17).

Buyer	Individuals	Family investors	LLCs	Non-LLC companies
Wells Built (*n*)	511	262	193	213
Average Lot Size [Acres-mean (SD)]	66 (SD = 87)	78 (SD = 101)	148 (SD = 175)	114 (SD = 158)
Surface Water Access (% of Parcels)	84.7%	77.9%	80.8%	74.2%
Average Groundwater Depth at Time of Drilling [Feet-mean (SD)]	101 (SD = 64)	119 (SD = 78)	154 (SD = 114)	115 (SD = 93)
Average Well Depth [Feet-mean (SD)]	465 (SD = 242)	535 (SD = 257)	736 (SD = 447)	645 (SD = 397)

Table 4 shows GAM results for predictors of well depth. The first model includes all buyer types, with individual owners as the reference group (*n* = 1179 wells, average well depth 557 feet, SD 334). The second model excludes individual buyers to compare the depth of new wells constructed by companies and family investors, with non-LLC companies as the reference group (*n* = 668 wells, average well depth 628 feet, SD 375).

**Table 4.** Generalized additive model (GAM) results estimating the association between well depth (in feet), buyer type, parcel characteristics, and year of well construction. Models were adjusted for latitude and longitude.

		Model 1: All buyer types *β* (std error)	Model 2: Companies and family investors only *β* (std error)
Intercept		−17 360*	−20 680
		(7492)	(10 660)
Buyer Type:^a^	Family Investors^b^	23.29	−49.97**
		(12.55)	(16.81)
	LLCs^c^	81.29***	10.97
		(15.03)	(18.30)
	Non-LLC Companies^d^	77.14***	*(Reference group)*
		(13.97)	
Irrigation District (Yes/No)		−38.28*	−60.16**
		(16.29)	(23.00)
Lot Size (Acres)		0.28***	0.24***
		(0.05)	(0.06)
Year of Well Construction		8.81*	10.53*
		(3.72)	(5.29)
Groundwater Depth (Feet)		1.26***	0.97***
		(0.16)	(0.23)
Crop Type:^e^	Orchard	−8.92	−13.32
		(11.45)	(15.94)
	Vineyard	−24.72	−6.91
		(18.21)	(25.22)
Adjusted R-squared		0.78	0.82
Number of Observations		1179	668
AIC		15 352	8759
Log Likelihood		−7597 (df = 79)	−4298 (df = 81)
Moran’s I (*p*-value)^f^		*p* = 0.33	*p* = 0.61

*Note:* Standard errors are reported in parentheses. *, **, *** indicates statistical significance at the 0.1, 0.05, and 0.01 level, respectively.

^a^ The reference buyer type for Model 1 is individuals. The reference buyer type for Model 2 is non-LLC companies.

^b^ All individuals and any associated trusts, IRAs, businesses, and other family investment vehicles containing the buyer’s last name and occurring on overlapping transactions were categorized as family investors (e.g. John Doe, Jane Doe, and the John and Jane Doe Family Trust).

^c^ The LLC category includes all remaining entries with ‘limited liability company’, ‘LLC’, or common misspellings occurring in the company name [19].

^d^ This category includes all non-individual entities which are not included in the LLC or family investor categories (e.g. corporations, partnerships).

^e^ The reference group for crop type for both models is row crops/pasture, in line with [71].

^f^ Moran’s I *p*-values > 0.05 suggest that the data do not show statistically significant spatial autocorrelation [75, 78].

Model 1 results indicate that compared to individual farmland owners, LLCs are associated with the construction of wells that are 81.3 feet deeper, and non-LLC companies are associated with the construction of wells that are 77.1 feet deeper, on average. Family investors’ new wells are on average 23.3 feet deeper than those drilled by individual owners. Model 2, which focused on companies and family investors, shows that compared to non-LLC companies, family investors are associated with the construction of wells that are 50 feet shallower, and LLCs are associated with the construction of wells that are slightly deeper (11 feet), on average. These results indicate that land acquisitions with one individual or family owner are associated with shallower wells.

## 4. Discussion

To our knowledge, this is the first statewide study analyzing the implications of farmland turnover for groundwater access. Our results show high farmland turnover rates across California, especially for acreage overlying groundwater; this dynamic is pronounced in critically overdrafted basins, where 23.5% of agricultural acreage turned over from 2003–17. The land market has substantially altered the composition of new groundwater rights holders over short timescales: by 2017, the overlying farmland acreage purchased annually by individuals decreased by half, and LLCs purchased one-third of all overlying acres sold annually. Together, these trends signal increasing corporate farmland acquisition, which is associated with the construction of wells 77–81 feet deeper, on average, than those drilled by individuals in the SJV basin. These findings raise concerns about groundwater sustainability and equity of access.

### 4.1. Overlying farmland turnover rates higher than expected

Studies suggest that the US farmland market is thin, but our findings in California challenge this assumption. National estimates suggest 0.5%–0.8% of farmland may turn over annually [79, 80], yet we estimate an annual turnover rate of 2.0% statewide for all farmland parcels over five acres, and higher still at 2.4% for overlying acreage (table 1). Previous work has raised concerns about high-profile transactions overlying groundwater in California [14, 42, 43]. Our results suggest that overlying land transactions are more common than previously assumed and identify numerous subbasins where rapid farmland turnover could have negative equity or sustainability impacts.

Land and groundwater rights are bundled in California. Therefore, overlying farmland turnover may alter multiple characteristics linked to groundwater extraction, such as farm size [15, 30] and the relative predominance of new owners, including those whose main income source is not from farming [24, 26, 28, 81]. Turnover may also affect the composition of groundwater management boards and the demographics of eligible voters in new groundwater management agencies [82–84].

Several drivers may explain high farmland turnover rates, such as changing commodity prices [85], sales by retiring farmers [23], the entry of new financial actors into the farmland market [14, 86], and changing production economics driven by current and forecasted water shortages [87], including under SGMA. Locally, shifting land–water management regimes may alter the importance of particular locations within basins: for instance, the establishment of groundwater markets under SGMA could increase the valuation of parcels with a high potential for artificial recharge [43]. Future research should examine turnover dynamics within subbasins (such as those areas suitable for managed recharge and located near water conveyance infrastructure), and potential factors driving land sales.

### 4.2. Increasing LLC acquisitions of overlying farmland

Recent case studies have noted the use of LLCs in groundwater-dependent agriculture in California [14, 43]. Our research demonstrates that, within the last decade, LLCs have become the primary entity acquiring overlying farmland in the state. These findings are consistent with studies documenting LLCs’ increasing agricultural land acquisitions elsewhere in the US [18, 19, 38]. Furthermore, we find that on average, LLCs bought 5.7 times more overlying land compared to individual buyers, suggesting increasingly concentrated holdings for this new corporate entity. These trends are most pronounced in critically overdrafted basins, where on average LLCs purchased almost 7 times more land than individual buyers.

The rapid increase in LLC land acquisitions is concerning because limited liability—the ability to protect members’ personal assets from corporate creditors—has been used as a mechanism to externalize risk at public expense (e.g. via water pollution) [88–90]. For example, pig farming operations have used multiple, interconnected LLCs to spread risk and shield assets in the event of a lawsuit against any one LLC [38]. In California’s Cuyama Valley, an LLC has attempted to produce land investment returns by proposing alterations to existing subbasin boundaries [14]. This emerging evidence underscores the importance of analyses of farmland ownership and large-scale transitions in the types of entities acquiring overlying rights.

### 4.3. Uneven well deepening: implications for groundwater sustainability and equity

Our results in the SJV suggest that, on comparable newly acquired parcels at comparable groundwater depths, companies and LLCs are associated with the construction of deeper wells than individuals and family investors. As groundwater wells deepen across much of the Western US [91], our analysis indicates that farmland acquisition and buyer type trends are important characteristics to track to assess potential sustainability and equity impacts.

Farmers may choose to operate an LLC for many reasons, including lower costs compared to incorporation [92] and more extensive shielding of assets [89, 90]. Considering the growing popularity of LLCs in agriculture [16], large-scale transitions in the legal liability structure of agricultural operations could potentially create adverse environmental impacts [38, 40]. Our models show no statistically significant difference between non-LLC and LLC corporate well depth for newly constructed wells. However, given substantial increases in the proportion of property purchased through LLCs, the difference in depth between LLCs and individuals or family investors drilling new wells merits more attention.

As groundwater becomes more important under a changing climate [93, 94], the trend towards deeper wells raises sustainability concerns [91, 95]. Since the lifespan of a constructed well is at least 25–35 years [96], deepening wells lead to infrastructural lock-in. Nationally, deeper wells on average draw from deeper water levels and therefore have greater pumping lift than shallower wells [57, 91]; pumping from increased depths increases the energy intensity of water supplies [91, 97]. New well construction may also harden water demand where it supports a switch towards permanent crops [15, 98]. Although well depth is not a proxy for pumping rates, groundwater over-extraction from deep wells may contribute to aquifer compaction and land subsidence [99, 100], and water extracted from deep semiconfined aquifers is more costly to recharge than that extracted from shallower aquifers [101]. Further, amid myriad SJV groundwater contamination sources, well construction and deepening may exacerbate and enable contaminant migration into deeper water sources via vertical cross-contamination [102], as well as encroachment on the defined bases of fresh water [103].

If implemented successfully, SGMA should ameliorate these undesirable impacts regardless of well depth, although its implementation success remains to be determined [55, 104, 105]. Indeed, groundwater levels are expected to continue declining under SGMA [55], which heightens equity concerns over well depth differences.

As companies are associated with the construction of deeper wells than individuals, they may be less likely to experience groundwater access disruptions related to future water level declines, thus exacerbating structural inequalities in groundwater access [5, 6, 55, 95, 96]. Our results show that new wells drilled by companies are, on average, around one-sixth deeper than those drilled by individual landowners, indicating an important difference in vulnerability to groundwater declines even in an aquifer-aquitard complex that is several thousand feet deep [70]. Small to moderate differences in well depth may confer important differences in vulnerability to water declines because SGMA establishes depth to groundwater as a regulatory standard [55], thereby codifying the centrality of well depth to groundwater access. Individual well owners’ vulnerability may be compounded for small and socially disadvantaged farmers who lack access to the capital needed to deepen or build new wells [57, 96, 106]. Further, while deeper wells reduce individual vulnerability to groundwater level declines, they may negatively impact neighboring users of shallower wells within the same aquifer unit, if their operation causes a localized drawdown of water elevations [57]. Although deep wells would not contribute directly to the dewatering of neighboring shallow wells if separated by an occluding layer [107], their operation can contribute to the overall downward-oriented vertical hydraulic gradients in the SJV basin [64, 108].

Our results provide a preliminary characterization of structural inequities in agricultural users’ vulnerability to future groundwater level declines. Although responsive to policymakers’ calls to investigate groundwater and farmland market interactions [109, 110], our analysis did not assess alternative strategies for ensuring water access, such as purchasing parcels with deep or recently-built wells, or procuring surface water rights, which may also vary across buyer type. Future research could extend the buyer typology developed in our analysis to: assess how additional owner characteristics (e.g. disparate financial and technological capacities, investment horizons, crop type choices over time, groundwater uses) influence groundwater access; discern policy and hydroclimatic effects on well owners’ drilling behaviors; and model spatially downscaled relationships between agricultural and domestic wells.

### 4.4. Limitations and future research

Due to data limitations, we are unable to identify farmland sellers (S2.3) or distinguish additional landowner characteristics that may affect farmland management strategies, such as whether landowners are first-time landowners [26, 28, 81], absentee landowners, or owner-operators [111, 112].

Of the 9.7 million transactions in ZTRAX that met transaction criteria (e.g. timeframe, arm’s-length), 1.7% did not conclusively join to any record in the assessment dataset. Given the large proportion of non-agricultural land transactions in California, many of these records may not refer to the sale of relevant parcels; thus, our numbers likely reflect a slight underestimate of the volume of transactions statewide from 2003–17 (S1.3). Additionally, we were unable to match 19.9% (*n* = 1152) of 2011–17 wells retained for matching at the parcel scale in the SJV basin; of these, 55.1% (*n* = 635) were missing an address, APN, or drill depth (S3.6).

This project focuses on assessing changes in overlying rights through the purchase of land and the construction of new groundwater wells. Existing groundwater rights and rights that do not require ownership of overlying property, such as prescriptive rights held by municipalities, are not addressed. Though our results are specific to California, they underscore the importance of analyzing overlying land transitions in regions where groundwater access is delineated by a landed private property rights regime, such as in Texas and Arizona [10, 113]. More broadly, our results highlight the importance of tracking changes in coupled land–water systems.

## 5. Conclusions

Our results suggest that overlying farmland is considerably more liquid in California than previously thought, affecting ongoing and future groundwater access patterns. The private land market has a substantial role in shaping groundwater access that needs to be further understood, particularly at a moment of large-scale transition in California groundwater governance. Who—or *what*—can access groundwater matters, as ownership of overlying land will become increasingly important in determining not only groundwater access, but also governance, recharge efforts, and the possibility of dominating nascent groundwater markets. Sustainability and equity concerns call for land ownership to be more explicitly considered in groundwater governance at local and regional scales.

## Data Availability

The San Joaquin Valley groundwater well dataset produced for this research is available at osf.io/uxhze/. Land transaction data cannot be made publicly available upon publication because they contain sensitive personal information. Transaction data suitably anonymized with identifiers and addresses removed are available upon reasonable request pursuant to the terms of our data use agreement; however, the study cannot be fully reproduced without the names, addresses, and identifiers, although data reproduction would be possible by manually compiling a similar dataset from publicly available county-level offices. Parcel data used for this research cannot be made publicly available upon publication because they are owned by a third party and the terms of use prevent public distribution. Information about data access is available at www.zillow.com/ztrax and www.digmap.com/platform/smartparcels/. All other datasets used in this study are publicly available from the references indicated.
